# Ocular Dominance and Its Association With Central Corneal Thickness: An Observational Study in Central India

**DOI:** 10.7759/cureus.41033

**Published:** 2023-06-27

**Authors:** Ppavani Gundreddy, Archana R Thool, Sri Lekha Rao, Lokesh M Vaishnav

**Affiliations:** 1 Ophthalmology, Jawaharlal Nehru Medical College, Datta Meghe Institute of Medical Sciences, Wardha, IND; 2 Community Medicine, Jawaharlal Nehru Medical College, Datta Meghe Institute of Medical Sciences, Wardha, IND

**Keywords:** dominant eye, specular microscopy, hole-in-card-test, central corneal thickness, ocular dominance

## Abstract

Background

The aim of this study is to determine ocular dominance and its association with central corneal thickness (CCT). These two parameters are of great significance in clinical practice; identifying the dominant eye helps in planning cataract surgeries, treatment of presbyopia, monovision correction, etc., and assessing the CCT helps in early diagnosis and management of keratoconus, glaucoma, contact lens-related complications, and dry eye.

Methods

A cross-sectional study that involves patients and volunteers who have come for a checkup to the ophthalmology department of the college hospital. Ninety patients were examined for this study within two months. The hole-in-card test is performed to determine the ocular dominance in people with normal and healthy eyes without any pathologies except refractive errors. Specular microscopy through a non-contact modality will be done to assess the thickness of the central cornea in both eyes. Statistical analysis was done using the paired t-test to compare the patient's eyes and the chi-square test, which helps us associate ocular dominance and CCT.

Results

Right eye dominance was seen in the majority of the participants (72.91%), whereas left eye dominance was seen in comparatively fewer participants (27.08%). The CCT of the dominant eye is found to be 520.40 ± 29.83 μm and that of the non-dominant eye is 524.40 ± 29.37 μm. A lower CCT in the dominant eye was seen in 83.33% of the subjects; 14.58% of them had a higher CCT in the dominant eye and 2.08% had the same CCT in both eyes.

Conclusion

From the observational study that has been made, the majority of the population shows right eye dominance. The CCT is relatively thinner in the dominant eye. About 80-85% of the examined people showed a thinner cornea in the dominant eye. But we cannot generalize that the eye with a lesser corneal thickness will be the dominant eye in all the cases, as a few cases have shown dominance in the eye with a thicker cornea.

## Introduction

In 1593, Giovanni Battista della Porta was the first to describe the phenomenon of ocular dominance. Ocular dominance is referred to as the eye preference of a person while trying to visualize something [1].

It is considered similar to right- or left-handedness (dominant hand), which always does not match with the dominant eye [2]. Research shows that a right-handed person is more likely to have right eye dominance, but there are a few cases where a dominant left-handed person has right eye dominance, which is referred to as cross dominance. Few people also show cross-dominance, which means the dominant eye and the dominant hand oppose each other. The theory of functional lateralization best explains ocular dominance, demonstrated by most paired organs like hands, legs, etc. [2,3].

In clinical practice, determining the dominant eye helps in planning cataract surgeries, treating presbyopia, selecting corneal profiles during refractive surgeries, choosing different types of intraocular lenses, and knowing patient satisfaction after visual or refractive correction [4]. Eye dominance is usually evaluated to correct vision in people who get monovision, where one eye is corrected for distant vision and the other for near vision. Monovision can be obtained through laser-assisted in situ keratomileusis (LASIK) surgery, contact lenses, and glasses. The dominant eye is usually preferred for setting distance vision during surgery. It is also useful in treating strabismus/amblyopia in children because the non-dominant/weaker eye will be affected if left uncorrected. The dominant eye will be covered or patched to strengthen the weaker eye [5].

Central corneal thickness (CCT) is a chief clinical factor that must be evaluated before any refractive surgeries, corneal transplantation, early diagnosis and management of keratoconus, glaucoma, contact lens-related complications, and dry eye. It should also be measured in patients with ocular hypertension, as it can determine the risk of conversion to glaucoma. Studies have found that people with thinner cornea have a greater risk of developing glaucoma [6].

In recent studies, ocular dominance was also tested to see its association with retinal fiber thickness and an inner plexiform layer of ganglion cells. It was noted that the thickness of the retinal fiber layer and the inner plexiform layer of ganglion cells was comparatively higher in the dominant eye [7,8]. Since most eye structures and cerebrum arise from a common embryonic derivative, there could be an embryological functional association between CCT and ocular dominance [9]. In this study, we are trying to analyze if there is any significant relationship between CCT and ocular dominance.

## Materials and methods

This cross-sectional study was conducted during August-September 2022 in a tertiary care institute in central India. On their first visit, all patients were given a detailed history taking and careful examination. The patients were examined for visual acuity using Snellen chart, intraocular pressure (IOP) using tonometry, and fundus examination using slit-lamp microscopy to check if they had any other underlying eye pathologies. 

Inclusion criteria included patients with bilateral non-pathological eyes and refractive errors. Exclusion criteria included patients with glaucoma, abnormal anterior and posterior segments, co-dominance in the hole-in-card test, and contact lens users.

Hole-in-card test

**Figure 1:**
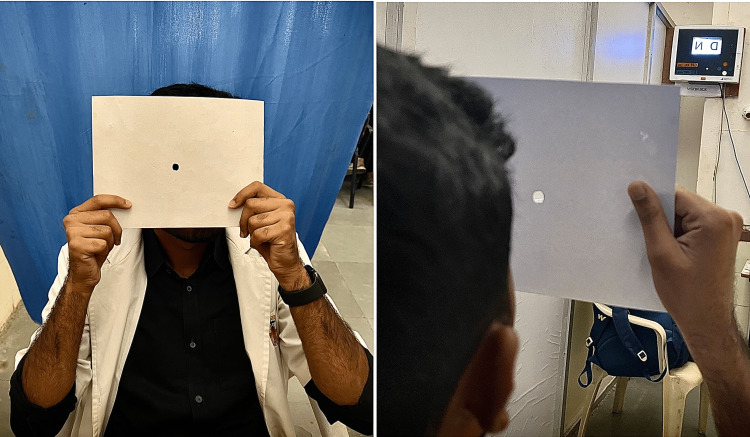
Determining ocular dominance by hole-in-card test.

This test is done to identify the dominant eye (sighting dominance). This is also called the Dolman method and is one of the most common methods used to identify the dominant eye in clinical settings [10,11]. The patient was instructed to hold a rectangular card with both hands. This card has a small hole in the center with a diameter of 4 mm and the patient should view a target at a distance of 6 m through the hole with both eyes open (Figure 1). First, they were told to close one of their eyes alternatively and view the target through the hole. Secondly, the subject was asked to bring the card close to the face by fixing the target until the hole is placed against one of the eyes, which is considered the dominant eye. The question of hand dominance can be avoided by using a hole-in-card test since both hands are used to hold the card in position [10,11].

Specular microscopy

**Figure 2:**
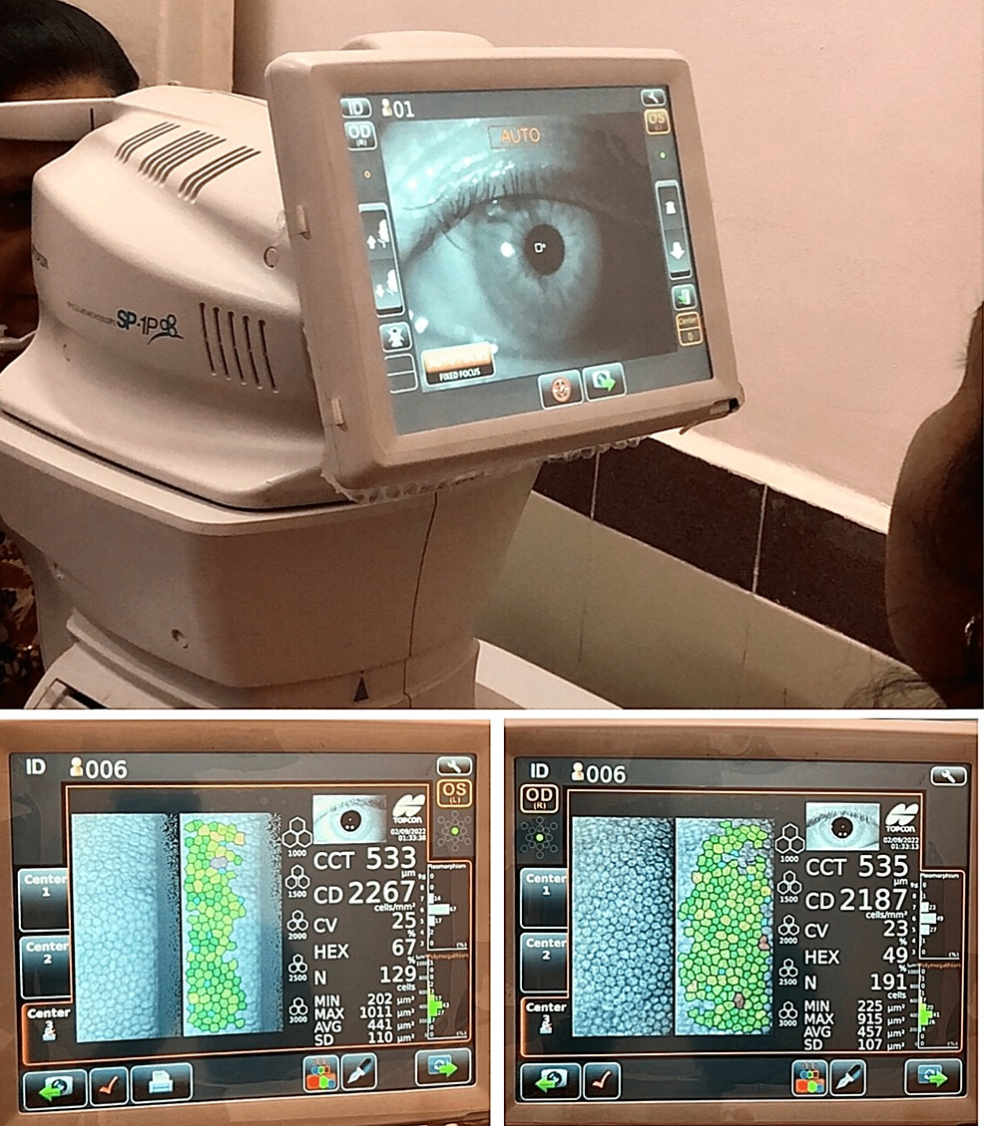
Measurement of CCT using a specular microscope. CCT: central corneal thickness.

This is done to evaluate the CCT of the eyes. CCT was measured through specular microscopy using a non-contact modality. It is a non-invasive diagnostic tool that helps assess the cornea and its endothelium [12]. Patients were asked to keep their heads still and visualize a fixation target in the microscope, and the results were generated automatically as soon as the center of the cornea was focused (Figure 2). The normal corneal thickness averages between 520 and 550 μm [13].

## Results

The patient's information was assessed and anonymity was maintained before performing the statistics. CCT was compared between the dominant and non-dominant eye with the help of the paired t-test. After assessing CCT in both eyes, the results were noted in comparison with the other eye and labeled as "high," "low," or "same." A chi-square test was done to find out the association between ocular dominance and CCT, i.e., to find out if the dominant eye and the non-dominant eye showed any association with the corneal thickness values. Statistically significant results were obtained (P < 0.05).

G power 3.1.9.7 was used to calculate the sample size using a confidence interval of 95%, an alpha probability of 0.005, an effect size of 0.3, and a power of 0.8. This makes up a sample size of 89, i.e., n=90.

The study was conducted on 90 members, which included volunteers and outpatients who came to the hospital. This includes both female patients (70.83%) and male patients (29.16%). The age of the study population is 23.77 ± 2.16, which ranges between 20 and 30 years and includes only bilateral eyes without any pathologies excluding refractive errors. Right eye dominance was seen in the majority of the participants (72.91%), whereas left eye dominance was seen in comparatively fewer participants (27.08%).

**Figure 3:**
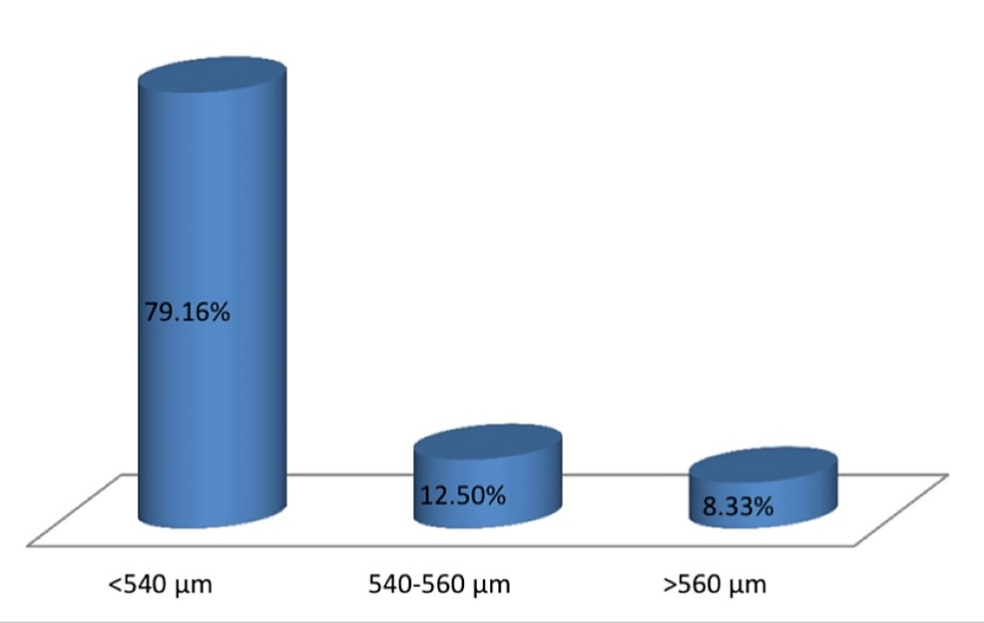
Distribution of CCT. CCT: central corneal thickness.

**Figure 4:**
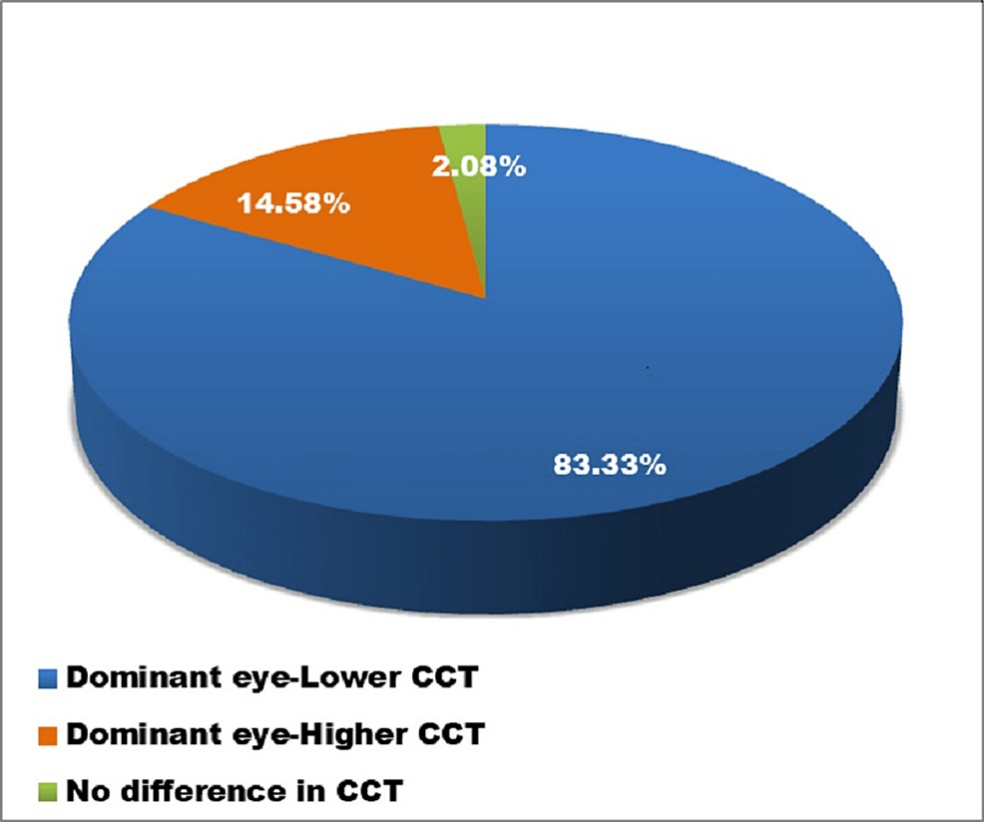
Relative ocular dominance and CCT. CCT: central corneal thickness.

The division of CCT in the eyes was examined into three categories (Figure 3). The CCT of the dominant eye is found to be 520.40 ± 29.83 μm and that of the non-dominant eye is 524.40 ± 29.37 μm. The lower CCT in the dominant eye was seen in 83.33% of the subjects; 14.58% of them had a higher CCT in the dominant eye and 2.08% had the same CCT in both eyes (Figure 4). As per the results obtained, there was no significant association between CCT and ocular dominance. Therefore, the eye with a lower CCT cannot be labeled as the dominant eye.

## Discussion

Ocular dominance

Hubel and Wiesel introduced the concept of ocular dominance columns in the 1960s [14]. According to this, two types of columns are found in the visual cortex of the occipital lobe. One is the ocular dominance column and the other is the orientation column. Our two eyes receive the information and combine it at the level of the primary visual cortex. The neurons receiving the input show a stronger response to stimulus from one eye compared to the other. Both eyes don’t function the same from a sensory and motor point of view, just like our hands [15,16]. The dominant and non-dominant eyes in an individual have relatively different functions; one performs the majority of seeing, whereas the other aids in visual activity. This basically means one eye leads the other or can fixate on something better with one eye than the other. It depends on the visual preference of a person to take input from one eye to another. This happens because each hemisphere of the eye controls the field of vision of the other half of the eye [16].

The majority of the population is usually right-eye dominant. Left eye dominance is prevalently noticed in certain conditions. Patients with Williams-Beuren syndrome and migraine show dominance in the left eye [17,18]. Ocular dominance can also switch between eyes depending on the task performed by the individual [19].

Determining the dominant eye would help us in the following ways:

1. In clinical settings, understanding ocular dominance can help us with better management approaches in treating certain visual conditions like monovision correction, certain eye conditions like strabismus (crossed eyes) and amblyopia (lazy eye), choosing an appropriate intraocular lens for cataract treatment, etc. [20,21].

2. To enhance the performance in sports and other activities.

In some sports, it is important to know the dominant eye for proper head positioning. Two such sports are golf and baseball. For good drives and shots, turning your head to a proper position to use your dominant eye is required. Cross-dominance has proven useful in sports like cricket. Most people who play certain sports like archery, darts, shooting, photography, etc., which require proper aiming, perform better with their dominant eye [22,23].

People using telescopes and microscopes regularly will be curious to know their dominant eye as it aids them in visualizing better. Ocular dominance is also found to be useful for Surgeons, especially for those who perform laparoscopic procedures [24].

Central corneal thickness

CCT is one of the most commonly measured parameters for an eye examination in clinical practice. Its measurement has been critical for patients undergoing corneal transplants and refractive surgeries and for assessing the risk of development of glaucoma [25,26].

The central cornea is only half as thick as the peripheral cornea, but it is more densely packed than the peripheral cornea. It also has five to six times more nerve fibers and collagen fibers than the peripheral cornea, which is loosely arranged. The central cornea is the first part that exhibits strong swelling and is more prone to hypoxia. Therefore, lactic acid accumulation occurs and results in corneal swelling, chiefly associated with contact lens wearers [27].

On investigation, it was also found that a gene named "POU6F2" is responsible for corneal thickness after conducting animal studies. After removing this gene in animal models, corneas became thinner [28]. CCT is usually under genetic control and also differs among various races of people (African Americans generally have thinner corneas) [29]. The CCT was found to be higher in male individuals comparatively. It was also found that CCT was thinner in people with myopia compared to people with hypermetropia [30].

The gold standard method to assess corneal thickness is ultrasound pachymetry. The cornea is first anesthetized, and a tonometer pen is then used by tapping on the eye's cornea to calculate corneal thickness on a digital display. Even though there are a lot of disadvantages with ultrasound pachymetry, including damage to the corneal epithelium due to the direct placement of the probe on the cornea, the requirement of topical anesthesia could alter the corneal thickness measurements. This can also cause corneal irritation and lead to infections due to the damage to the corneal epithelium. The results also depend on the operator, making them less reliable. It also helps in determining corneal edema, evaluating patients with keratoconus, preoperative assessment in corneal surgeries, and postoperative health of a corneal transplant. Devices that are used for determining corneal thickness are based on two principles. The first principle is ultrasound-based, which is used by old devices like pachymeter. The second is the optical method, which is used by the latest devices like specular microscopy [31,32]. 

Many newer modalities such as optical coherence tomography, screening section pachymetry, and pentagram are being used by many clinicians. These methods provide the advantage of non-contact and objective determination of the thickness of the cornea [31,32].

## Conclusions

From our study, the CCT was predominantly thinner in the dominant eye compared to the non-dominant eye. Dominance in the right eye was found in the majority (70-75%) of the population with normal eyes (i.e., without any pathologies). There is no proper reason to explain why most of them had thinner cornea in the dominant eye. Relative CCT is not associated with the dominant eye because few patients showed higher corneal thickness in the dominant eye, unlike the majority of the population, and few people also showed no difference in the thickness of the cornea in the dominant and non-dominant eyes. So, we cannot always label the eye with a relatively thinner cornea as the dominant eye.
